# “We choose this CV because we choose diversity” – What do eye movements say about the choices recruiters make?

**DOI:** 10.3389/fsoc.2024.1222850

**Published:** 2024-03-07

**Authors:** Sayaka Osanami Törngren, Carolin Schütze, Eva Van Belle, Marcus Nyström

**Affiliations:** ^1^Department of Global Political Studies, Malmö Institute for Studies of Migration, Diversity, and Welfare, Malmö University, Malmö, Sweden; ^2^Brussels Institute for Social and Population Studies (BRISPO), Vrije Universiteit Brussel, Brussels, Belgium; ^3^Lund Humanities Lab, Lund University, Lund, Sweden

**Keywords:** hiring discrimination, diversity, vignette study, eye-tracking, decision-making, survey experiment

## Abstract

**Introduction:**

A large body of research has established a consensus that racial discrimination in CV screening occurs and persists. Nevertheless, we still know very little about how recruiters look at the CV and how this is connected to the discriminatory patterns. This article examines the way recruiters view and select CVs and how they reason about their CV selection choices, as a first step in unpacking the patterns of hiring discrimination. Specifically, we explore how race and ethnicity signaled through the CV matter, and how recruiters reason about the choices they make.

**Methods:**

We recorded data from 40 respondents (20 pairs) who are real-life recruiters with experiences in recruitment of diverse employees in three large Swedish-based firms in the finance and retail sector in two large cities. The participating firms all value diversity, equity and inclusion in their recruitment. Their task was to individually rate 10 fictious CVs where race (signaled by face image) and ethnicity (signaled by name) were systematically manipulated, select the top three candidates, and then discuss their choices in pairs to decide on a single top candidate. We examined whether respondents’ choices were associated with the parts of the CV they looked at, and how they reasoned and justified their choices through dialog.

**Results:**

Our results show that non-White CVs were rated higher than White CVs. While we do not observe any statistically significant differences in the ratings between different racial groups, we see a statistically significant preference for Chinese over Iraqi names. There were no significant differences in time spent looking at the CV across different racial groups, but respondents looked longer at Polish names compared to Swedish names when presented next to a White face. The dialog data reveal how respondents assess different CVs by making assumptions about the candidates’ job and organizational fit through limited information on the CVs, especially when the qualifications of the candidates are evaluated to be equal.

## 1 Introduction

Racial and ethnic diversity in European countries is ubiquitous today. While 20% of the EU population has a migratory background (i.e., being foreign-born or having one or two foreign-born parents), these individuals are facing discrimination across countries and sectors (Zschirnt and Ruedin, 2016; Auspurg et al., 2019). Among EU countries, Sweden has one of the highest rates of foreign-born people (20%). Sweden’s diversity is also marked by so-called second-generation Swedes that make up 7% of the population^1^. While being one of the most diverse countries in the EU, Sweden has also one of the highest rates of labor-market segregation (Quillian et al., 2019). For example, statistics on foreign-born people and their descendants published by Eurostat shows that despite the educational attainment being similar among the majority and minority population, the percentage of persons employed within an elementary occupation is much higher among those who are second generation immigrants compared to natives^2^. Possible explanations for this continued disadvantage in the second generation include weaker intergenerational transferability of skills by immigrant parents (Borjas, 1992; Borjas, 1993; Rooth and Ekberg, 2003), segregation or neighborhood effects (Tasiran and Tezic, 2007) and remaining unexplained components, known as ‘ethnic penalty’ that reflect different types of discrimination (Heath and Cheung, 2007; Rafferty, 2012; Zwysen et al., 2021).

Numerous field experiments and correspondence tests conducted in different European countries indeed confirm that persons with a migratory background are discriminated against in the labor market due to aspects such as their foreign-sounding names (e.g., Zschirnt and Ruedin, 2016; Ahmad, 2020; Thijssen, 2020). To counter discrimination, there is also evidence that persons with migratory background “whiten” the CVs through modifying their foreign-sounding names (Ruedin and Van Belle, 2023). While research in Europe has established that there is evidence of discrimination toward persons with a migratory background, there are a couple of aspects that are less clear. First, the process and mechanisms of how discrimination is perpetuated, pertaining to the question of why discrimination persist. Second, even though studies that address the different functions of ethnicity and race on the CVs are emerging (e.g., Di Stasio and Larsen, 2020; Fibbi et al., 2022; Polavieja et al., 2023), how race and ethnicity as a separate factor may play a role in discrimination still needs to be clarified.

While correspondence experiments are the gold standard to measure the extent of discrimination, what happens between the moment a researcher sends out CVs and the callback is still a black-box. This article aims to shed light on this process, by examining the way recruiters view, select and reason their CV selection choices, as a first step in unpacking the patterns of hiring discrimination. We focus specifically on understanding how native-born persons with migratory background may experience discrimination in the labor market, due to their racial and ethnic background. Combining a vignette study with eye-tracking and dialog, we acquire multiple data points in an experimental setting. By simulating the initial phase of the hiring process (CV selection), we explore the following questions: *How do race and ethnicity affect the ratings of the CVs? How do race and ethnicity affect how recruiters scrutinize a CV* (i.e.*, how long they look at different sections of a CV*)*? How do recruiters reason their choices of the CVs?*

As a method, eye-tracking can measure attention, where people look and how they move their eyes, which has been related to novel or cognitively problematic stimuli. For example, in a recent study which explores the utility of eye-tracking in understanding how we construct an social understanding of race, participants paid visual attention to subject’s hair (straight or curly) in determining whether the subject is Black, White or mixed-race (Sims et al., 2024). Studies also shows that visual attention is actively involved in preference formation (Shimojo et al., 2003; Parnamets et al., 2013).

The current study is an exploratory study which focusses on selection discrimination, i.e., which CVs are screened in or out, and not the entire hiring process where hiring decisions are the outcome of a long process involving multiple actors. However, through the qualitative data acquired through the dialog between two recruiters discussing their choices, we tap into the consensus making process, how selection is motivated and how individuals interpersonally rationalize CV selections.

We targeted companies that are actively engaged in looking at their recruitment practices with their internal and external diversity, equity and inclusion consultants. While more and more organizations are starting to consider diversity in hiring, especially in today’s tight labor market, there are still limited numbers of studies that look at these companies explicitly. There is evidence that diversity initiatives in organizations do not necessarily lead to achieving diversity and equity for various reasons. The most eminent argument is that diversity initiatives often are used to shield individuals and organizations, and signal that they are not discriminating. Indeed, a correspondence experiment from the United States shows that companies that explicitly include a pro-diversity statement in their job advertisements exhibit the same amount of discrimination as companies without such statements (Kang et al., 2016). Moreover, diversity trainings targeting individuals do not address deeper organizational or structural discrimination that exist in society (Dobbin and Kalev, 2022; Zheng, 2022). Our study targeting companies that are actively addressing discrimination in hiring practices therefore contributes to understanding the role and impact of individual recruiters’ choices and consensus making processes when the organizational culture and structure are favorable and supporting diversity and taking positive actions in countering discrimination.

The concepts of diversity, race and ethnicity are debatable and contested concepts. Diversity can be broadly understood as any observable and non-observable difference or similarity between people (Gottardello, 2019; Gross-Gołacka et al., 2022). Diversity initiatives at organizations are defined as “the implementation of one or more practices aimed at improving the workplace experiences and outcomes of groups that face disadvantage in society” (The Economist Economist Intelligence Unit, 2009). In this article we specifically focus on diversity in terms of race, ethnicity and gender (Dobbin and Kalev, 2022; Zheng, 2022). Race and ethnicity, both understood as socially constructed categories, are defined in numerous and contesting ways by different scholars, especially between Europe and North America (Banton, 2015; Morning and Maneri, 2022; Osanami Törngren and Suyemoto, 2022). In this article, we understand race as socially constructed system of hierarchy and power where phenotypes and other visible differences are deployed as boundaries of groups (Lentin, 2020; Osanami Törngren and Suyemoto, 2022), and ethnicity as cultural origin and heritage. Here, we operationalize both race and ethnicity as analytical categories to understand how CVs representing native-born Swedes with and without a migratory background, i.e., how White Ethnic Swedes, White non-Ethnic Swedes and Non-White Swedes, are treated at the initial stage of the hiring process. We operationalize race through photos representing Black, Middle Eastern (MENA), Asian and White faces, and ethnicity through names.

## 2 Discrimination in hiring and the Swedish context

The latest report from Statistics Sweden (2023) utilizing data from The Labor Force Survey’s (AKU) supplementary survey 2021 shows that among those of working age (20–64) 4.4% of the native-born Swedes are unemployed, while the number is 18.4% for foreign born persons. There is a significantly larger unemployment gap between the native and foreign born in Sweden than in the EU. The unemployment rate for the foreign-born was 8.6 percent and for the domestic-born 4.8 percent in the EU (SCB, 2023). The higher level of unemployment among the foreign-born population is sometimes explained through the level of skills and education and the time needed to “catch up” to the native population, which includes social network building to launch a career (Bevelander and Irastorza, 2014). The aforementioned report indeed shows that the gap is the largest among those who do not have secondary education, where 68% of the native born had an employment while only 49% of the foreign-born had an employment (SCB, 2023). The report also shows that 14.3 percent of foreign-born women and 10.8 percent of foreign-born men indicated a lack of language skills as an obstacle to getting a suitable job (SCB, 2023). The remaining differences in employment rates after accounting for human capital or demographic characteristics are known as the ‘ethnic penalty’ (Rafferty, 2012), which encompasses different types of discrimination. Studies from various contexts such as Switzerland and the UK proof the existence of significant ‘ethnic penalties’ after accounting for factors such as human and social capital and other employability criteria (e.g., Auer and Fossati, 2019; Zwysen et al., 2021). Indeed, in the aforementioned report, discrimination due to foreign background was reported as an obstacle to employment by 5.7 percent of women and 5.3 percent of men (SCB, 2023) Earlier research reports in Sweden indicate more specifically that foreign-born persons from Asia (including the Middle East) and Africa experience discrimination the most. Once employed, research shows that occupational segregation exists as well. Occupational segregation in Sweden is shown to be both vertical (foreign-born population face difficulties in achieving higher job positions within the organization) and horizontal (foreign-born population is concentrated in low-paying jobs) (Diskrimineringsombudsmannen, 2012; Wolgast et al., 2018; Wolgast and Wolgast, 2021).

While the gap in employment between natives and foreign-born is undeniable in Sweden, the disparity in employment level between natives and children of immigrants, the so called ‘second generation’, also continues to be prominent in Sweden (Behtoui, 2006; Hammarstedt and Palme, 2012; Dahlstedt, 2015). Here the question of “time needed to catch up to the native population” is challenged. Indeed, research based on register data, behavioral data of employers, and correspondence testing shows that, even though skills and work experiences gained in Sweden counter labor market discrimination positively, factors such as foreign sounding names (especially Arabic sounding names) and foreign backgrounds (such as Somalia or Iraq but not Poland) do affect employers hiring decisions negatively (e.g., Agerström and Rooth, 2009; Carlsson, 2010; Rooth, 2010; Vernby and Dancygier, 2018), independent of qualifications. In addition, the aforementioned research shows that prejudice exhibited by the managerial employers create an ethnic wage gap in Sweden, and employers with more negative attitudes toward ethnic minorities act on such prejudice in the initial hiring stage (Rooth, 2010; Carlsson and Rooth, 2012; Carlsson and Rooth, 2016).

Moreover, Sweden’s reliance on employers’ and unions’ decision and their oversight of hiring processes, combined with the lack of a monitoring system (e.g., statistics on possible disparities related to race and ethnicity) due to its color-blind approach, may also contribute to the high level of hiring discrimination (Quillian et al., 2019). Color-blindness in Sweden is powered by the antiracist discourse of individual choices and equal opportunities for all independent of ethnic and racial background (Osanami Törngren, 2019; Hübinette, 2023), which contributes to the belief in meritocracy (Doane, 2017). In fact, reports specifically examining Afro-Swedes and their experiences in labor market do show that longer education for Afro-Swedes is not always advantageous, but rather result in higher unemployment (Wolgast et al., 2018).

From the previous research in Sweden, it can be inferred that employers may discriminate against some categories of applicants but not others in recruitment decisions for reasons not wholly related to qualifications. The results from a number of correspondence experiments indicate indeed that Swedish-born persons of migrant background may face discrimination. A meta-analysis of field experiments of racial discrimination in hiring conducted in nine countries in Europe and North America shows that Sweden, together with France, has the highest level of hiring discrimination toward non-White minorities (Quillian et al., 2019). Carlsson and Rooth (2007) found that persons with Middle Eastern sounding names receive 50% lower callback for a job interview as compared to those with native Swedish sounding names. In addition, it is shown that ethnic discrimination in Sweden is larger when the labor market is tighter (Carlsson et al., 2018a). Moreover, even though there is no general neighborhood signaling effect, there is a significant interaction effect between the neighborhood and foreign background. Fictious job applicants with a foreign background faced significantly lower call-back rate if they signaled living in a stigmatized neighborhood with lower income and higher unemployment rate, compared to job applicants with a foreign background who signaled living in a more privileged neighborhood (Carlsson et al., 2018b). Finally, there is evidence that ethnic and racial discrimination intersects with gender. For example Arai et al. (2016) find through a field experiment that men with Arabic sounding names face stronger discrimination than Arabic women (Arai et al., 2016). Another study confirms that ethnic discrimination affects foreign-named men the most and especially within male dominated occupations (Bursell, 2014). A recent study on how social perception of warmth and competency depending on intersections of ethnicity, gender, sexual orientation and age shows the complexity of how one identity can offset negative and positive perceptions depending on the stigmatized category (Strinić et al., 2021).

## 3 Theoretical framework and hypotheses

From the existing research on hiring discrimination in Europe – and Sweden in particular, we expect the fictitious CVs of majority White-Swedish candidates to be preferred to CVs with a different race or ethnicity. This brings us to our first set of hypotheses:

*H1a:* CVs with a non-White picture (race) will receive a lower rating than CVs with a White picture.

*H1b:* CVs with a non-Swedish name (ethnicity) will receive a lower rating than CVs with a Swedish name.

The seminal work of Bertrand and Mullainathan (2004) did not only show that Black sounding names were significantly less likely to receive a callback for a job interview, but also that the CV quality (e.g., Ivy League education) of Black candidates did not impact callback in the same way as the CV quality of White candidates. This begs the question whether part of the observed differences in callbacks can be explained by differences in attention given to the CVs of different candidates. Bartoš et al. (2016) measure differences in attention given to CVs with a majority and minority name using a field experiment and find indeed that recruiters reduce efforts when reading a minority CV. A recent study using cookie data tracking the search behavior of recruiters on a large online job platform in Switzerland, on the other hand, did not find evidence that recruiters spend less time on the CVs of minority candidates (Hangartner et al., 2021). Eye-tracking provides a direct measurement of how much attention recruiters pay to (different elements of) CVs.

Eye-tracking research on how employers scrutinize CVs is sparse. We therefore look at research in different fields, including marketing, psychology, and educational sciences to hypothesize how employers might scrutinize CVs and how this might differ between majority and minority candidates. We distinguish between how long respondents look at the CV, and how much they spend looking at specific areas in the CV (i.e., the total dwell time within an area of interest [AOI]).

Research from psychology shows that respondents normally spend more time looking at things that they like in a given visual material and visually focus more on the options that respondents ultimately choose (e.g., Maughan et al., 2007; Ghaffari and Fiedler, 2018). There are also studies that shows that participants spend more time looking at different attributes that are presented when facing difficult choice tasks (e.g., Ryan et al., 2018). Moreover, when presented with two visual stimuli (faces), respondents may start by looking at the two stimuli equally but gradually shift more attention toward the face respondents chose (Shimojo et al., 2003). Therefore, we hypothesize that a longer total dwell time will be correlated with a more positive rating, i.e., more favorable to choosing the CV as top candidates (H2).

Concerning where respondents look, research also shows that own-race faces are recognized more accurately than other-race faces, a finding known as the ‘own-race bias’ (Meissner and Brigham, 2001; Wan et al., 2017). Several studies have explored eye-movements and the own race advantage in face recognition (Hayward et al., 2008; Goldinger et al., 2009; Hayward et al., 2013; Gonzalez and Schnyer, 2019). Moreover, studies show how other race-, or other gender-faces receive initial attention, while own-race and own-gender faces are viewed longer (Lovén et al., 2012), or how stereotypes may affect attention to other races (Donders et al., 2008). Furthermore, a broad literature in the field of cognition has shown that people look longer at text and/or pictures if there is an inconsistency between both (e.g., in our case a Swedish name combined with a non-White face) (see for example Schüler, 2017).

Eye-tracking is an emerging method to be incorporated into understanding labor market discrimination. To the researchers’ knowledge, there is only a handful of studies including Lahey and Oxley’s (2016, 2018) studies exploring the potential of eye-tracking for understanding the hiring process. Our study is inspired by Lahey and Oxley’s studies which utilize randomized CVs and eye-tracking, exploring the effects of race on employment discrimination over the life cycle in the US context. Their study shows that respondents’ time viewing the entire CVs, as well as their viewing patterns, do not vary by the type of CVs. However, respondents spent less time on CVs for younger Black candidates compared to other groups. The authors conclude that this disparity is due to respondents using negative heuristics or engaging in taste-based- discrimination against young black applicants. A conjoint study using eye-tracking by Jenke et al. (2021), examining how respondents process information presented in the conjoint surveys found a clear correspondence between where respondents look and the attributes of the profile that are considered to be important.

Based on the above, our hypotheses are:

*H2:* A longer total dwell-time will be correlated with a higher CV rating.

*H3a:* A longer dwell-time will be recorded on the face AOI for non-White face CVs.

*H3b:* A longer dwell-time will be recorded on the name AOI for non-White face CVs.

*H4:* A longer dwell-time on the specific face and name AOIs will be correlated with a perceived discrepancy between the race and the ethnicity.

Apart from the hypotheses above, exploring how CVs are screened using the quantitative data we gather through the vignette study, we analyze qualitative data acquired through a dialog between two respondents discussing their choices with the theoretical understandings of how recruiters assess person-environment fit (Schneider, 1987; Van Vianen, 2018). Person-environment fit has two sides, the employer’s needs and the employees’ desires and preferences. If we focus on the employer’s perspective, person-job fit is about whether the job applicant profile fulfils the qualifications that are required for the specific job, while person-organization fit addresses the compatibility of the individual to the organization in terms of personality, culture and values (Kristof, 1996; Kristof-Brown et al., 2005). Recruiters and managers evaluate potential employees through a number of overlapping processes involving assessment of person-environment fit, through the evaluation of acceptability (organization-fit) and suitability (skills and qualifications) of the candidates. Moreover, discrimination may be present in both the assessment of suitability and acceptability, but it may be more visible and influential in the evaluation of acceptability (Jenkins, 1986). Assessment of person-environment fit and why it is important might also vary individually (Piasentin and Chapman, 2006; Nolan et al., 2016), but research shows how recruiters tend to focus more on the person-organization fit especially in interview settings (Chuang and Sackett, 2005), and people rely and trust their intuitive expertise that they would be able to evaluate the complex characteristics of an job applicant (Highhouse, 2008). Experimental studies comparing how respondents treat Swedish and Arabic applicants (signaled by name) do show that ethnic minority candidates prompted recruiters to focus more on questions related to person-culture fit (Wolgast et al., 2018).

Related to the above, Rivera (2020) suggest in her work about employer decision making an “employers screening theory.” This theoretical framework puts forth that employers select based on (1) competence, which includes education and experience, status signals and social ties. Additionality they select based on (2) status beliefs and stereotypes and (3) social closure, which includes feelings of dislike for particular groups and desire to preserve opportunities based on group membership. In order to assess these different elements of potential candidates and make callback decisions, recruiters use the available candidate information discerned from the CV. The model set forward by Derous and Ryan (2019) stipulates that when job-related personalized information is limited (as is the case in our experiment), recruiters might revert to categorization based on non-job-related stigmatizing applicant information. This categorization increases the risk of screening discrimination (Derous and Ryan, 2019). Studies in Sweden also show that working with a structure procedure of recruitment lead to an improvement in the selection of more competent applicants, however there was no clear results whether structured recruitment counter discrimination (Wolgast et al., 2017).

Our study deploys standardized CVs with limited job-related personalized information and the process of CV selection is unstructured due to the limited information given to the respondents about the job the candidates are supposed to be recruited for. Therefore, we assume that respondents will engage in a range of conversation about person-job and person-organization fit, but we expect that this conversation will include emphasis on their organizational effort to diversify their employees.

## 4 Data and methodology

### 4.1 Respondents

A total of 40 respondents were recruited in three companies located in Sweden, two in finances and one in retail. All three are international companies but two of them are Sweden-based. Contact with the companies was initially made by a third party which consults these companies with Diversity, Equity and Inclusion initiatives^3^, and the researchers were introduced to the contact persons in each company responsible for the human resource department who could further facilitate the recruitment of respondents. All companies communicated clearly that they are working actively to increase diversity, equity, and inclusion in their organization. The contact persons in each company were instructed to provide respondents in pairs, who should be in a similar position within the organization to avoid hierarchical relations affecting the decision-making process. All respondents worked directly with recruitment or had up until recently worked with recruitment. Given that we are only involving companies and respondents working with diversity initiatives in recruitment, our respondents may not be representative for how recruiters and hiring managers make choices in Sweden.

### 4.2 Data collection procedure

**Figure 1:**
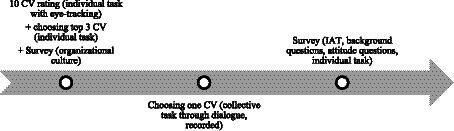
Study design.

Data was collected at the locations of the companies (in Malmö and Stockholm) between December 2022 and January 2023. Respondents from the same company took part in the data collection as a pair in identical set ups in two different rooms with no direct sunlight, but otherwise well lit. The experiment consisted of three parts generating quantitative and qualitative materials for analysis. The first and the last part of the study were based on individual tasks. The first part of the study consisted of two sections. The first section was the vignette study using eye-trackers where respondents were given 5 min to view 10 randomly selected fictious CVs on a computer screen and give an assessment of how likely it is that the person on the CV will be called to an interview (1–7 scale). Simultaneously, respondent’s direction of gaze (where they looked on the computer screen) was recorded by an eye tracker. Respondents went through a trial round where three example CVs were presented as a means of orienting themselves to the task. In the second section, respondents selected the three top candidates and also answered questions about organizational culture (Podsiadlowski et al., 2013) and the racial composition of their co-workers. The second part of the experiment was a 10-min dialog between each pair, where they discussed the three candidates they each selected. Their task was to agree on one candidate to call for an interview. The dialog between the respondents was video and audio recorded over the digital platform Zoom. The third part of the experiment was an individual web-survey, including the Implicit Association Test, explicit attitudinal questions (McConahay, 1986; Akrami et al., 2000; Rooth, 2010) and background information.^4^ All three parts were conducted in two separate rooms with identical settings where respondents were sitting alone in the room (see Figure 1).

This paper will solely focus on the results acquired in the first and the second part of the study, more specifically the rating of the 10 CVs and the selection of the top candidate in the dialog. We are analyzing these different components separately to gain a holistic understanding of what respondents see in the CVs, how they rate the CVs, and create consensus about which CV is the best candidate. In the following we describe the vignette study, eye-tracking and dialog design and our data analysis more in detail (Table 1).

**Table 1:** Respondents.

	*N* = 40
**Gender**	
Male	11
Female	29
Average age	37.3 (24–63)
**Recruitment experience**	
Less than 5 years	19
6–10 years	10
More than 10 years	11
**Self-identification (race)**	
White/European	36
Non-White	4
**Self-identification (ethnicity)**	
Swedish	33
Non-Swedish	7

### 4.3 Vignette study with eye-tracking

Respondents were given the following instruction:

You are going to be reviewing applicants for an entry level economist position that does not require any prior specialized skills. The CVs are standardized and have already gone through a pre-selection process. The candidates are all born and raised in Sweden. They have graduated in 2021 from a university in Sweden with a degree in Business and Economics and applying for work. You have 5 minutes to review the candidates (scroll back and forth). Please rate each of the 10 CVs how likely you are to invite the person for an interview (1=Will not call to an interview – 7=Will call to an interview).

After completing the 5-min CV screening, respondents were presented with the 10 faces and names that they saw on the CV together with the rating that they gave earlier and were asked to choose their top 3 candidates.

The fictitious CVs differed in a predefined number of factors, as depicted in Table 2. Each CV was first assigned a picture. In order to keep the set of evaluated CVs as realistic as possible, 70% of CVs were assigned a White face, and 30% of CVs were randomly assigned a non-White face.

**Table 2:** Vignette levels.

Factors	Vignette levels
Face (race)	White, Middle Eastern, Asian, Black
Gender	Male, Female
Name (ethnicity)	Swedish, Polish, Iraqi, Chinese, Eritrean
Motivation*	One sentence motivation
Education	BA degree or MA degree in Economics, with or without distinction
Thesis*	Information on the BA/MA thesis
Experience*	One up to 7 years of experiences within sales
Skills*	Microsoft Office or Microsoft Excel

*Experience and skills are not included in the vignette hierarchies in the analysis.

Similar to Di Stasio and Larsen (2020) field experiment, these fictious CVs aimed to measure the impact of race therefore White faces were assigned either Swedish or Polish names (60/40), and the non-White faces were assigned either Swedish names or the names that matches the racial background (50/50, e.g., Asian face and a Chinese name). We used facial photos from two different facial databases^5^ and modified the color of the clothing to all black to avoid distraction. Faces that are used in the survey were chosen following a thorough evaluation of the norming data that are attached to the facial databases. In addition, an extensive robustness check survey was performed, re-evaluating the attractiveness of the faces in the Swedish context and determining how Swedish the faces were perceived to be. The names were selected in consultation with colleagues of the signaled ethnicity and were independently tested in order to determine whether they (i) conveyed the right ethnicity, (ii) the correct gender, (iii) were associated with a young person (all our fictitious candidates were recent graduates), and (iv) did not signal attractiveness, social class or religiosity.^6^ The full list of names included in the experiment is provided in Supplementary Appendix. Apart from the face and name, each CV was randomly assigned an education level (BA or MA with or without distinction) and varying years of experience in the retail sector (0–7 years). The random assignment of these factors and levels allows us to causally test the importance of each factor, as these are independent by design (Auspurg and Hinz, 2014).

Apart from these factors, we added a number of additional elements to make the 10 CVs distinct and realistic. These factors that did not put the CVs into hierarchies were the one sentence motivation (e.g., I am searching for an exciting career as an economist with the recently acquired degree in Business and Economics; Recently completed an education in Business and Economics, and I am looking for opportunities to start my career and apply my knowledge), thesis topic (e.g., Determinants of Changes in Capital Structure on the Nordic Market, Examining the Effectiveness of Discounted Cash Flow Models) and skills (Microsoft Excel or Microsoft Office). All aspects of vignette were tested through robustness check survey to be perceived as neutral, and the CVs were tested through a pilot study.

While the respondents were reviewing the 10 randomly selected CVs, their eye-movements were recorded. Binocular eye-tracking data (gaze position on the computer screen) was collected with the Tobii Nano (Firmware v. 2.41.0-f2dc56e) at 60 Hz using the Titta toolbox (git commit v. d6d9715) Niehorster et al. (2020) and the Tobii SDK (v. 1.7.0). The Tobii Nano is a remote one camera, video-based eye tracker capable of both dark and bright pupil tracking, and records data in the screen’s coordinate system, where (0, 0) corresponds to the upper left corner of the screen and (1, 1) the bottom right corner of the screen. Stimuli were presented with PsychoPy 2022.2.3 on a Samsung S22E450F 22 Inch (1,920 × 1,080 pixels, aspect ratio 16:9) monitor with a refresh rate of 60 Hz. Data were collected with two identical setups in windowless rooms. The respondents viewed the stimuli from 65 cm. Titta’s default 5-point calibration followed by a 4-point validation were performed at the beginning of each recording. As recommended by Holmqvist et al. (2022), the quality of the eye-tracker data was reported, and was, across respondents and eyes given by the validation in the Titta toolbox; accuracy: 0.63 degrees (SD = 0.28); S2D-RMS precision: 0.13 degrees (SD = 0.08). About 3.5% (SD = 4.5%) of recorded samples were reported as invalid by the eye tracker, e.g., due to blinks. To restrict head movement, respondents were asked to place their elbows on the desk in front of them, put the hands together, and rest their chin on their hands (Figure 1).

**Figure 2:**
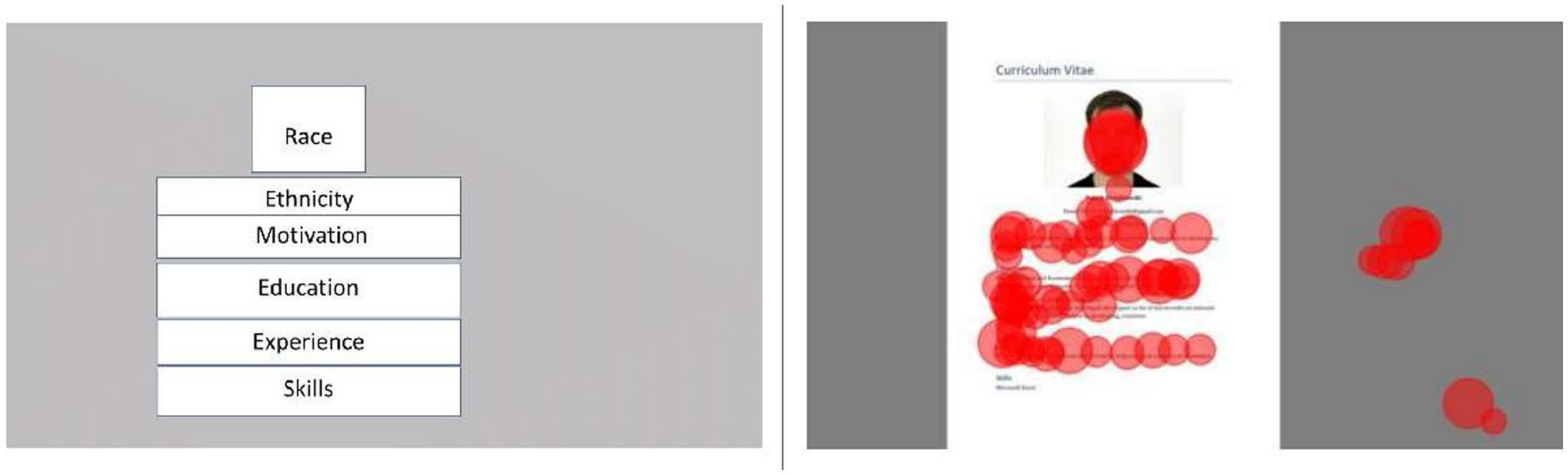
AOIs (left) and example of CV with eye-tracking data (right).

From the eye-tracking data, we have defined areas of interests (AOIs, see Figure 2, left) consisting of different parts of the CV that correspond to the vignette levels: Face, contact information including name, a short motivation to why they apply to the job, education including topic of thesis, experience and skills. The total dwell time in milli-seconds on AOIs was computed as the sum of all gaze samples falling within a certain AOI. The AOIs were defined manually and are shown in Figure 2.

First, the rating of the CVs and the eye-tracking data were analysed separately. The rating of the CVs was analysed by comparing means between two groups in form of a *t*-test and by comparing means between more than two groups in form of a one-way ANOVA analysis, followed by a Tukey HSD test to detect differences between group means.^7^ The eye-tracking data was analysed through comparing how long respondents looked at the CV in total and at the different AOI’s in particular (their so-called dwell-time) and comparing it across groups with a *t*-test and a one-way ANOVA as described above. After the two separate analysis, we examined if the CV rating is correlated with the dwell-time of the different AOI’s on the CV with the Pearson’s correlation coefficient (Agresti, 2018).

Each circle represents one fixation, and the diameter of a circle is proportional to the fixation duration. Here, only the CV is shown, but during the actual recording additional elements were shown to rate the CV and go to the next/previous CVs. Fixations on the grey area represent fixations associated with such elements. Fixations were detected for illustration purposes using the I-2MC algorithm with default settings (Hessels et al., 2017).

### 4.4 Dialog

After completing the vignette study and answering questions about their organization, respondents were connected via a Zoom meeting. In the dialog, each pair was presented with the top three candidates of their choices and was given 10 min to discuss and come to a decision which candidate should be called to an interview. First the moderator (one of the authors) presented the top three candidates one by one through share screen (minimum 4 and maximum 6 CVs) and then instructed the respondents to start discussing. They were also told that the moderator was not going to take part in the conversation, but that they could tell the moderator which CV they would like to be displayed in the Zoom meeting. They were alerted 1 min before the time was up. Most of the pairs took the whole 10 min, but nine pairs concluded before the time was up. Two pairs carried out the dialog in English and the rest had a dialog in Swedish. All dialog were transcribed and translated into English.

The transcribed and translated dialogs were then coded and analyzed in NVIVO (Jackson and Bazeley, 2019). Analysis of the dialog was deductive and the coding strategy emerged from the understanding of assessment of organizational fit (Jenkins, 1986; Jewson and Mason, 1986; Schneider, 1987). Codes also correspond to the CV content (diversity, motivation, education, thesis, work experience and skills) which were then further broken down in detailed coding (e.g., BA, MA, degree with distinction, total years of working, work description). We should note that the codes were assigned and identified to the content by one of the first authors based on her interpretation of the dialog, in dialog with other authors.

## 5 Results

### 5.1 Vignette study

In order to test our hypotheses (H1-4) and provide more insight into how recruiters treat different CVs, we first analyse the findings from the vignette study. We consider two important outcomes: (i) the CV ratings (i.e., the likelihood that a CV is invited for an interview); and (ii) the dwell-time on the overall CV and specific AOIs.

#### 5.1.1 Difference in the CV ratings by race

**Figure 3:**
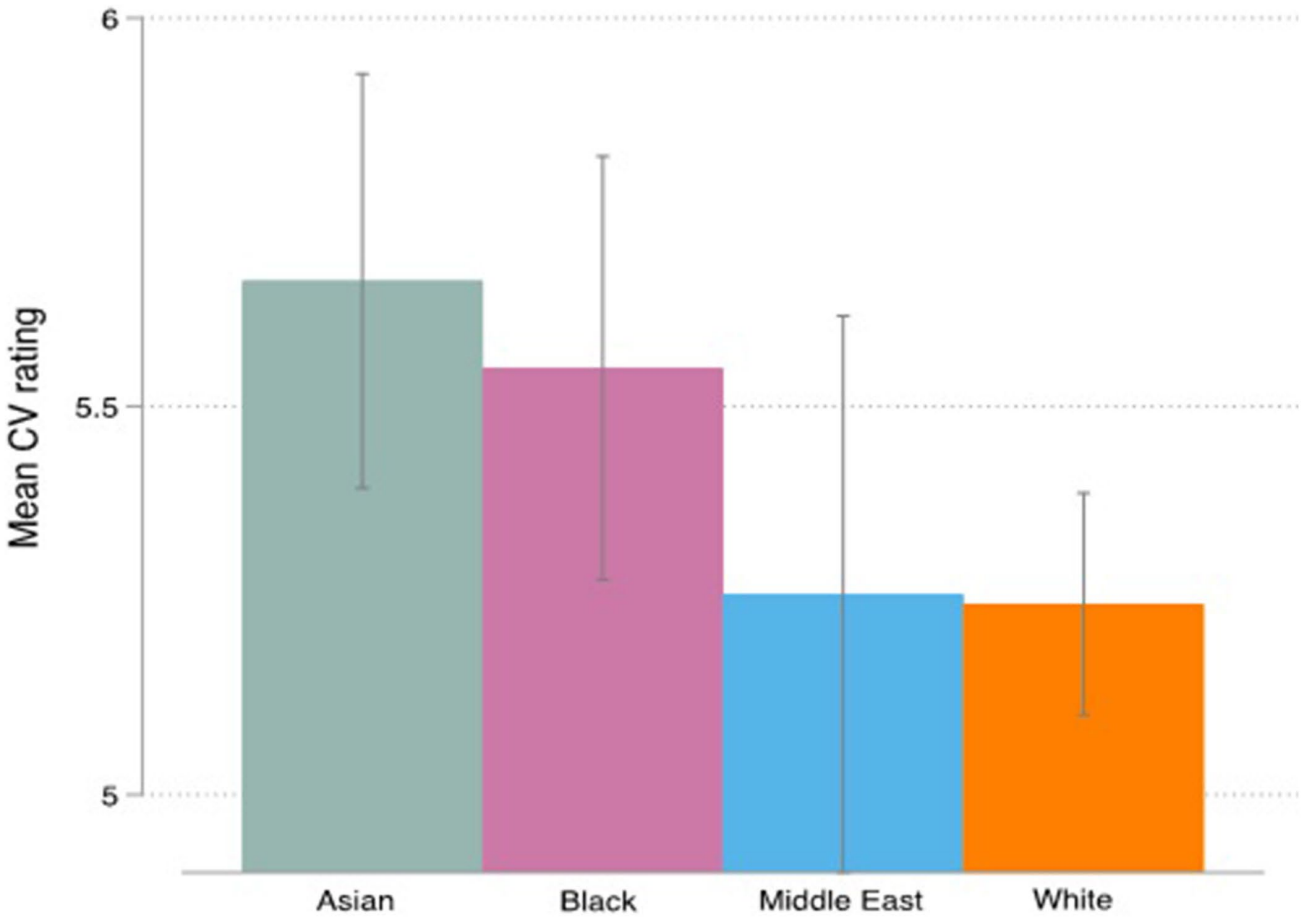
Graph bar mean CV rating by race with CI’s.

In total 200 CVs were tested in the vignette study (See Supplementary Appendix for the number of CVs per race and ethnicity that were tested) (Figure 3).

Figure 3 above summarizes the means of CV rating across racial groups showing the highest rating for Asian CVs (5.66) followed by Black CVs (5.54); Middle Easter CVs (5.25) and the lowest rating for White CVs (5.24). A one-way ANOVA revealed that there was no statistically significant difference [*F* (3,3) = 2.63, *p* = 0.050]. Tukey’s HSD Test for multiple comparisons found that there was no statistically significant difference between any of the four groups. To directly address our hypothesis (H1a), we tested whether the CV rating would differ between white and non-white faces. For this analysis we combined all non-white faces (Middle Eastern, Asian, Black) and compared them to the White category. There was a significant difference (two-sample t-test) in the CV rating between white (*M* = 5.24, SD = 1.24) and non-white faces (*M* = 5.49, SD = 1.33); *t* (534) = 2.14, *p* = 0.032, with CV’s showing White faces having a lower mean rating. We performed the same test for respondents first rating of the CV and respondents last rating of the CV (since respondents were allowed to go back and forth between CV’s and adapt their rating) and the result of the *t*-test shows a non-significant difference in the CV rating between white and non-white faces for the first rating but a significant difference for the last rating. These findings reject our first hypothesis on the effect of race on the CV rating (H1a).

#### 5.1.2 Difference in the CV ratings by ethnicity

**Figure 4:**
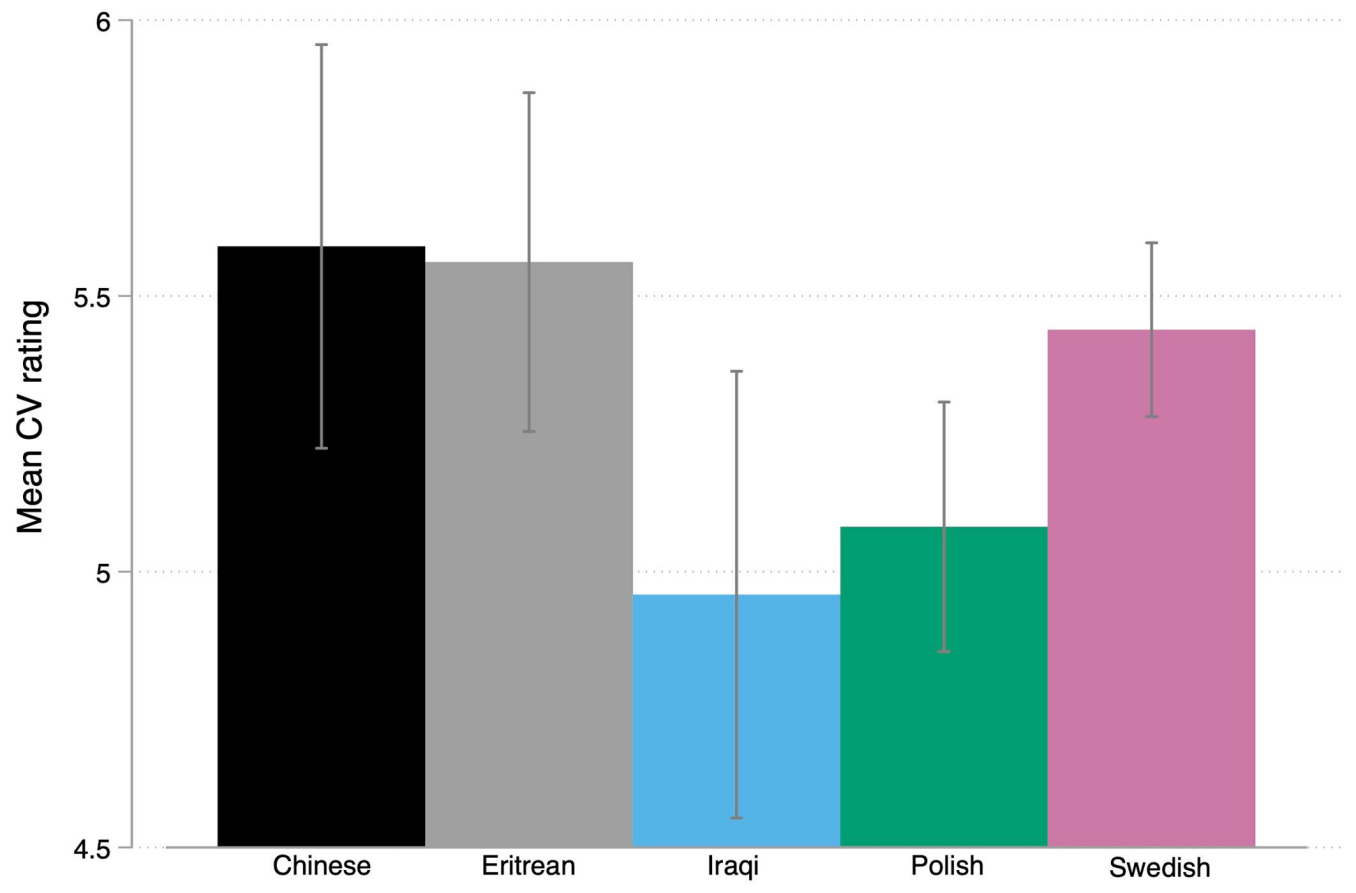
Graph bar mean CV rating by ethnicity with CI’s.

Figure 4 summarizes the means of CV rating across ethnic groups showing the highest rating for Chines names (5.58) and Eritrean names (5.56) followed by Swedish names (5.4); Polish names (5.0) and the lowest rating for Iraqi names (4.9). A one-way ANOVA was performed to compare the effect of CV rating on all five ethnic groups as signaled by the names (Chinese, Eritrean, Iraqi, Polish, Swedish). There was a statistically significant difference in CV rating between at least two groups [*F* (4,4) = 3.45, *p* = 0.008]. Tukey’s HSD Test for multiple comparisons found that the mean value of CV rating was significantly different (*p* < 0.05) between Chinese (*M* = 5.58) and Iraqi names (*M* = 4.95). There was no statistically significant difference in mean between the other groups. In order to test our hypothesis (H1b) we once more grouped Swedish and non-Swedish ethnicities. The *t*-test shows a non-significant difference between Swedish names (M = 5.4; SD = 1.28) and non-Swedish names [*M* = 5.2; SD = 1.31; *t* (534) = −1.8, *p* = 0.07]. As a result, we have to reject our hypothesis H1b. Nevertheless, there seems to be some evidence of ethnic hierarchies in hiring preferences.

#### 5.1.3 Difference in the CV ratings by gender

We also tested if the CV rating would differ between male and female candidates. A two sample *t*-test was performed to compare the CV rating in CV’s showing male and female faces. There was no significant difference in the CV rating between male (*M* = 5.38, SD = 1.34) and female faces [(*M* = 5.28, SD = 1.26); *t* (534) = −0.891, *p* = 0.373].

### 5.2 Eye-movement

In this section, we analyze the eye-movement data gathered during the vignette study. We first look at total dwell-time and whether this differs by race, ethnicity or gender of the CV before looking at the dwell-time on specific AOI’s, testing our hypotheses 3 and 4.

#### 5.2.1 Dwell-time difference by race

In a next step we investigated if the total CV dwell-time would differ between White and non-White faces. A two sample *t*-test was performed to compare the CV dwell-time showing White and non-White faces. There was no significant difference in the CV dwell-time between White (M = 14461.68, SD = 9612.03) and non-White faces [(*M* = 14153.35, SD = 9830.61); *t* (538) = −0.35, *p* = 0.721]. A one-way ANOVA was performed to compare the effect of CV dwell-time on all four racial groups. The figure above summarizes the means of dwell-time across racial groups. The one-way ANOVA revealed that there was not a statistically significant difference in CV dwell-time between the groups [*F* (3,3) = 0.35, *p* = 0.789].

#### 5.2.2 Dwell-time difference by ethnicity

**Figure 5:**
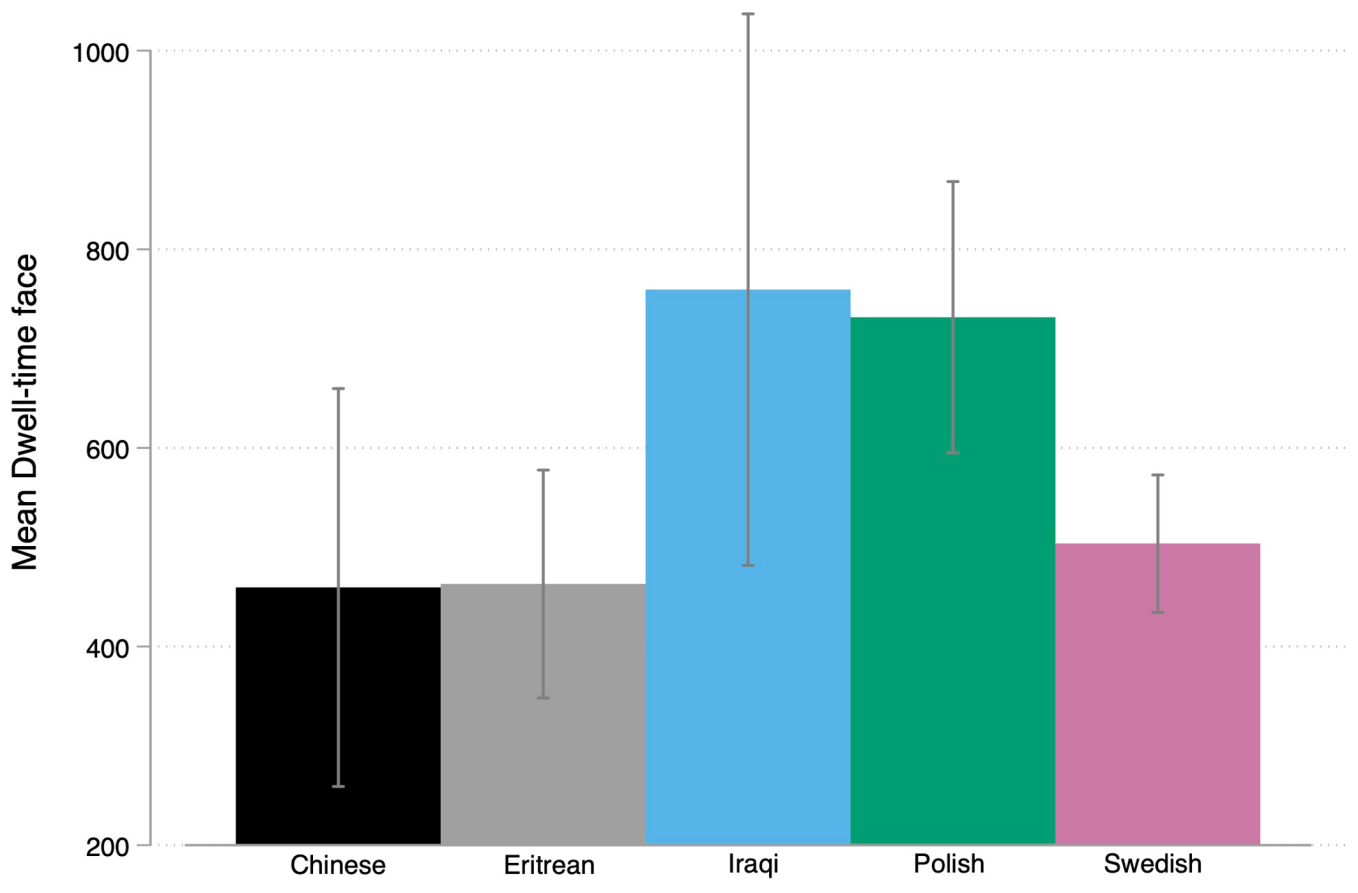
Graph bar mean dwell-time face by ethnicity with CI’s.

A one-way ANOVA was performed to compare the effect of CV dwell-time on all five ethnic groups (Figure 5). The one-way ANOVA revealed that there was not a statistically significant difference in CV dwell-time between the groups [*F* (4,4) = 1.54, *p* = 0.1903].

#### 5.2.3 Dwell-time difference by gender

We also tested first if the CV dwell-time would differ between male and female candidates. A two sample *t*-test was performed to compare the CV dwell-time in showing male and female faces. There was no significant difference in the CV rating between male (*M* = 14557.43, SD = 9900.77) and female faces [(*M* = 14125.89, SD = 9464.94); *t* (534) = −0.517, *p* = 0.605].

#### 5.2.4 Dwell-time specific AOI by race

**Figure 6:**
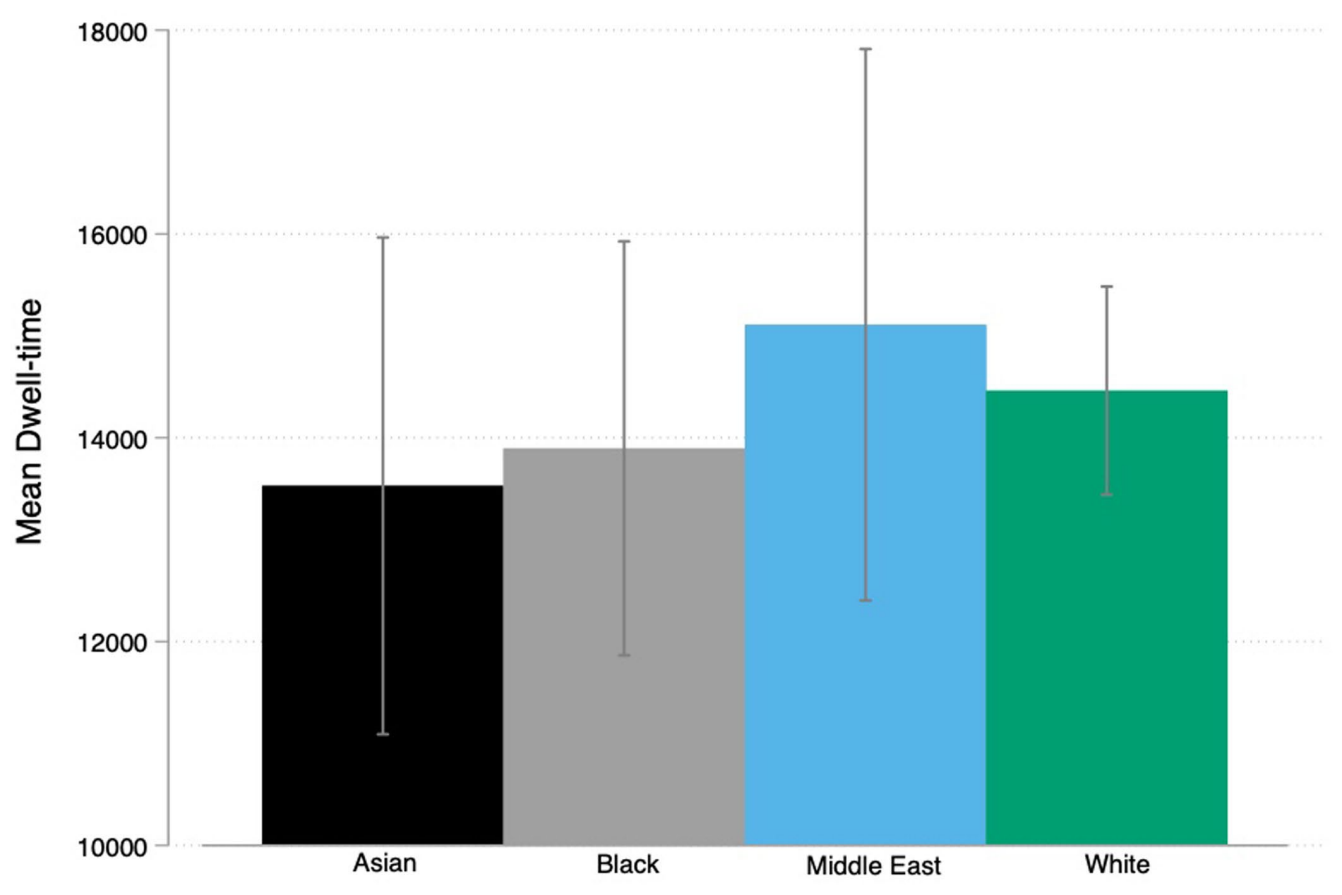
Graph bar mean dwell-time by race with CI’s.

Next, we tested if the different AOI’s (face, name, education, experience, skill) differ between white and non-white groups and the four specific racial groups with the help of a t-test and an ANOVA (Figure 6). None of the results showed a significant difference between groups for the specific AOI’s. We performed the same tests for respondents first rating of the CV and respondents last rating of the CV with no significant differences between the groups for both, the first and the last rating. This finding rejects our hypothesis 3a, as there is no significant difference between dwell-time on specific face AOI for different racial groups.

#### 5.2.5 Dwell-time specific AOI by ethnicity

Additionally, we tested if the total dwell time on different AOIs varied due to the ethnic differences (names). The figure above summarizes the means of dwell-time on the face AOI across ethnic groups. A one-way ANOVA was performed to compare the effect of CV dwell-time on all five ethnic groups. The one-way ANOVA revealed that there was a statistically significant difference in face dwell-time (how long respondents look at the face on the CV) between two groups [*F* (4,4) = 4.13, *p* = 0.002]. Tukey’s HSD Test for multiple comparisons found that the mean value of face dwell-time was significantly different between Polish (*M* = 731.58) and Swedish names (*M* = 503.56) with a *p* value under 0.05. There was no statistically significant difference in means between the other groups. We performed the same tests for respondents first rating of the CV and respondents last rating of the CV with the same results.

This finding confirms our hypotheses (H3b and H4) that if the CV shows an inconsistent element, in this case a White face with a Polish name instead of a Swedish name, then the respondent will view this specific AOI longer. Indeed Polish names themselves, as in Chinese, Iraqi or Eritrean names, may attract more visual attention since it may require a longer cognitive processing of the foreign-sounding name or foreign words in general (e.g., Mishra, 2009; Crossley et al., 2014). Eye-tracking research on reading also shows that fixation is longer for less familiar words (Rayner, 1998). However, in our analysis we are observing difference in the dwell-time on the face (race AOI) rather on the name (ethnicity AOI). This is in line with literature on cognition that shows that people look longer at a text or picture if there is an inconsistency between them (Schüler, 2017). One of the ways to interpret this result may be that when people in Sweden see a White face they expect to see a Swedish name and the respondents visual attention gathers to the CVs with Polish names that are unexpected.

#### 5.2.6 Vignette study and eye-movement

In a last step we correlated the CV rating with the CV dwell-time. First, we correlated the CV rating with the total dwell-time, resulting in no significant correlations. Second, we correlated the CV rating with the AOI specific dwell-time, resulting in no significant correlations. We performed the same tests for respondents first rating of the CV and respondents last rating of the CV with the same results.

This means that our hypothesis (H2), that is in line with previous research, that a longer dwell-time should be correlated with a higher CV rating, is not confirmed.

### 5.3 Dialog

100 CVs in total were selected as a top three candidate, and no pairs chose the exact same three candidates (see Supplementary Appendix). 10 pairs shared one common candidate (discussed 5 CVs in total), 4 pairs had two common candidates (4 CVs) and 4 pairs had no overlapping candidates (6 CVs). Through the dialog, CVs with racially Black profile were chosen the most (*N* = 9) next to White CVs (*N* = 7), and then Middle Eastern CVs (*N* = 3). Only one Asian CV was chosen. When it comes to ethnicity, 7 Eritrean, 2 Iraqi and 3 Polish CVs were chosen, and no Chinese CV was chosen (see Supplementary Appendix). 12 CVs were female, all CVs except one have both MA and BA, and a large majority of them with distinction in at least one of the degrees. Working experiences varied; the majority of the CVs had ongoing work experience or currently finished employment, with few exceptions of CVs that had gap years from the time of the data collection (December 2022 to January 2023).

**Figure 7:**
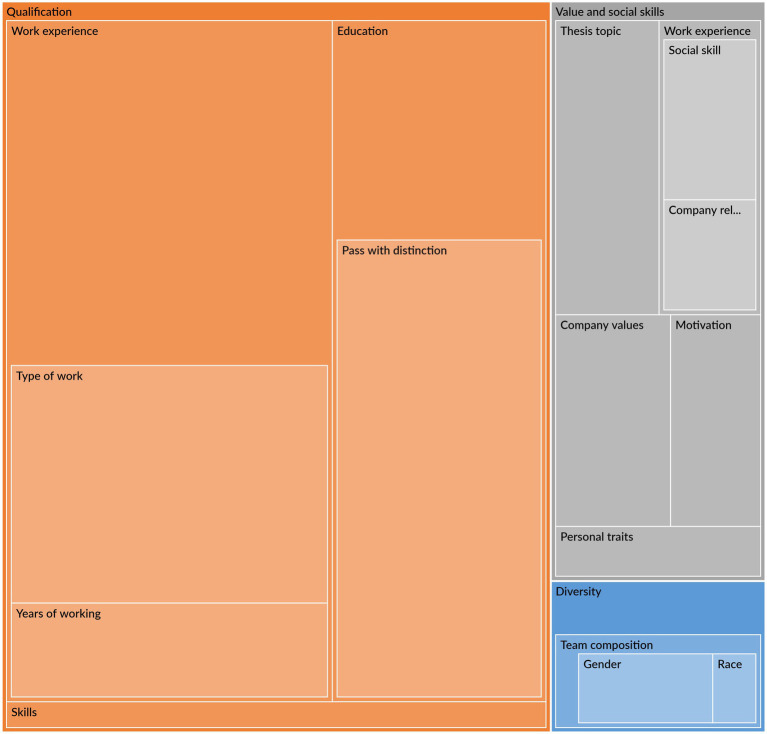
Map of coded utterances.

Analyzing the dialog, the reasoning around the choice of candidate centered around several themes that assess how the individual candidate fit into the organization, which we categorized in three groups for the purpose of our analysis: qualification, values and social skills and diversity. Examples of the utterances from the respondents in different themes are presented in Supplementary Appendix. Despite the respondents being recruited from companies that are engaging actively in the question of diversity in hiring, surprisingly little time were spent addressing racial, ethnic and gender diversity of the candidates in relation to the suitability of the candidate (see Figure 7 which shows the share of coded utterances). Of a total of 754 coding, 72% of the dialog was related to the qualification of the candidate, 22% on values and soft skills, and 6% of the time addressing diversity. The majority of the respondents stated either how the CVs are all similar and equal or referred to the initial instruction that said, “all candidates are qualified for the position.” The interesting point of exploration is how respondents differentiated what they perceived as equally qualified candidates.

We identified three pairs basing the choice purely on person-job fit (qualification), two pairs purely on assessment of person-organization fit (values and social skills), while the other 10 pairs focused on an interconnected assessment of interrelated aspects of person-job and organization fit, and four pairs specifically discussed the aspects of diversity. While the qualification and the diversity of the candidates could be read straight forwardly from the CV, social skills and other aspects that can assess person-organization fit were interpreted through the choice of topic of the thesis, the type of previous work experience (e.g., customer service, sales, desk-work or work in stores) and the one sentence motivation that was stated on the CV.

#### 5.3.1 Assessing person-job and organization fit

Recruiter A and their pair made the final choice based purely on the level of education. They had 6 CVs to start with and while the total years of work experience varied from a year to 7 years, only 1 CV had distinctions on both BA and MA degree. Recruiter A spelled out the instruction that “all are qualified” and explained to their pair:

Recruiter A: I knew all kinds of things, like dimensions you can weave in like yes, but he works at X company for 4 years with customer support while someone else works with something else somewhere else. **But it becomes a question of value, kind of which I don't think is relevant. It is not possible to quantify and compare unless someone happened** to work as an economist.

Recruiter B and C had 5 CVs with varying educational level (2CV had only BA degree) and varying years of work experiences to discuss. While Recruiter B pushed for another candidate based on the social skills that B assumed through the type of work experience, C convinced B that they should pick the only candidate with distinctions on both BA and MA degree instead of looking at what kind of work experience the candidate has.

Recruiter B: I probably do lean more maybe to [Black Eritrean Female] or, she [White Polish Female]. […] we can check [Middle Eastern Iraqi Female] again. And here, too, I think that **she has only worked with customers via call cases via phone and chat, so she hasn't met customers or met people in the same way?**

Recruiter C: Why do you need it, if you are going to work as an economist?

Recruiter B: Because you must work in a team, you will meet people and you have stakeholders and then I think that it is a good experience, that you have not only worked with sales [on the phone]. I think of it [meeting customers] as a social competence.

Recruiter C: Yes, or she has been very motivated in her studies and thus invested in it during that period and that doesn't say anything about, it [the kind of work you had] doesn't say anything about her social skills.

As illustrated by the quotes above, we observe diverse understandings of how individual may fit to the organization from the standardized CV with limited information. Moreover, assessment of previous work experience turned into a question of not only person-job fit but also varying understandings about person-organization and culture fit. For example, recruiter D assessed how the candidate may fit to the company through the choice of thesis topic and experience of working in service.

Recruiter D: I think everyone has met the requirements. Interesting experience and such. But the candidate has written about “cash flow models”. It is very relevant to economics. And a good experience also from working in service.

Even the pairs that had chosen the CV with the highest education and the longest working years among the CVs that were discussed sometimes reasoned the choice not solely based on the qualification. What the dialog reveals is the process of assessment of person-organization fit through the assumptions made on the generic one-line motivation statement, the thesis topic or the types of work experience that was stated on the CV. For example, recruiter E compared the topic of the thesis and measured how the interest may fit to what the company does. The pair had 5 CVs that all had a MA degree, whereof one had a distinction on both BA and MA. All except one candidate was currently working. Recognizing that it was difficult to make a choice, the breaking point became what is the best fit for the company, which was assumed through the topic of the thesis:

Recruiter E: Hazard models. It also feels very national economic and the concepts […] “the purpose of this thesis is to examine the determinants of corporate failures from the pool of accounting and market driven variables". Yes, accounting and market driven variables, it is closer to what we do. Maybe if you think analyst, compare to the other thesis looking at “a new index of welfare systems”.

For other respondents, how the candidate may fit into the organization was measured through what kind of work experience the candidate had or in combination of both. Below recruiter F leans in their subjective evaluation toward different work experience:

Recruiter F: The only thing that really separates them is that one has worked with sales and the other has worked with support and then I would react more to sales. Then I would go for grades and [experiences in] sales.

Despite the profile being the most qualified in terms of education (MA degree) and work experience (working 2017–2022), one pair, assumed the personality of the candidate through the generic motivation sentence and work experience, which became the breaking point of choosing the candidate to call for an interview.

Recruiter K: I also thought that [Black Male] stood out a bit, partly this with like “I am looking for opportunities to start my career and apply my knowledge that I have acquired”. He felt like he had some confidence that he shows. He has sort of gained knowledge here that he can sort of get into and then he has worked with customers, phone chat, solving customer's problems. That's why I thought it was [the best candidate].

Another pair, recruiter L and M, on the contrary, given the statement that all are qualified, based their final choice purely on their assumption about the personality, choosing a candidate with a BA degree with work experience (2018-current). While the candidate chosen had the longest years of work experience, there were other candidates with MA degrees with distinction.:

Recruiter L This one [thesis] was very relevant, so I feel that she focuses a little more on the Nordic market. […]

Recruiter M: What did you choose her too? Because I thought, it [motivation] was “exciting career”. She is excited. She studied and she still working, and she has skills. I think it feels like she ticks all the boxes.

#### 5.3.2 Consideration of diversity in organization

Despite the favorable evaluation of non-White CVs on the vignette study, the majority of the pairs did not explicitly address gender, racial and ethnic diversity in their dialogs. Moreover, diversity was mainly spoken of in terms of gender diversity (six pairs), with only three pairs specifically considering the racial and ethnic diversity of the co-workers in the organization when discussing the CVs. What is attention grabbing is the cases where the pair below decides to go for the CV that represents diversity rather than what the pairs seem to agree as the most suitable for the position. The pair considered the White Swedish Female candidate and the Black Eritrean Female candidate to be equally strong candidates. They both have an MA degree and the only difference on the CV is the signaled race and ethnicity, and that the Black Eritrean Female notes that she is currently working (since 2019) while the White Swedish Female indicated working experience between 2017–2020. The conversation below clearly shows that what is decisive for them was the aspect of diversity. The exchanges are fast as the 10 min is running out, and there are doubts but no reasoning in between, except a statement “we choose inclusion”.

Recruiter G: She [White Swedish Female] also has an MA and she has longer work experience than the one in the middle [Black Eritrean Female] has purely objectively but it also depends if they are two equally strong candidates, then maybe we would have taken the other [Black Eritrean Female] based on an inclusion, that you want diversity in the workplace. It depended but we don't know.

Recruiter H: No, if we take based on that [qualification], I would say, [White Swedish Female].

Recruiter G: Who should we take? [White Swedish Female] or [Black Eritrean Female]? Then we should take her in the middle [Black Eritrean Female].

Recruiter H: Should we take her? Yes, I think we decide on the middle one [Black Eritrean Female] because we choose inclusion.

A similar exchange can be observed in another pair. The pair chose a Black female CV that they discuss as with “extremely small margins” differ on the paper:

Recruiter I: But if this were to be a real recruitment process, then in this context, when we have come this far, we would think, what does the team look like? What does diversity in the team look like today? How can we widen diversity in the team where these people will land and then it is most likely that we would have taken [Black Eritrean Female, BA with distinction and MA, work experience 2019-2021].

Recruiter J: […] I think all these candidates have relevant thesis and relevant academic background. There are extremely small margins in that way, but if you go by academic merits, perhaps [White Male, BA and MA with distinction, work experience 2017-2020] feels sharper on paper as it is.

Recruiter I: Yes, then one would like to add a team composition perspective to this, I feel, but we don't have that information, but we can assume that it is like that.

Recruiter O: Exactly.

Recruiter J: Or should we add a diversity perspective, for example […]if we were to take a diversity focus here, it would be [Black Male, BA with distinction and MA, work experience 2020-2021] or [Black Female].

## 6 Discussion

This paper adds to the growing evidence showing that ethnicity and race affect CV ratings. Moreover, we show that ethnicity also affects how employers scrutinize a CV. In addition, we demonstrate that respondents’ reason about these choices through the acceptability and suitability of the candidate. The paper is based on a vignette experiment in combination with eye-tracking and dialog. We tested a number of hypotheses in this paper. Relating to the eye-tracking data, we proposed that a longer total dwell-time will be correlated with a higher CV rating. This hypothesis was not confirmed as our analysis showed no significant correlation between dwell-time and CV rating. This was a surprising result, given that Lahey and Oxley (2018) could show in their study that respondents spent less time on CVs for younger Black candidates compared to other groups, which resulted in a preference of younger white applicants to younger black applicants.

Next, we proposed that a longer dwell-time on specific AOIs will be correlated with aspects of CVs that are unknown or unexpected. While we do not find an effect of lesser known aspects (i.e., other-race or other-ethnicity effects), we do find a significant effect of unexpected aspects on the CV. Indeed, our analysis showed that the mean value of the face AOI dwell-time was significantly different between Polish and Swedish names, in this case indicating a surprising element, when a White face is presented with a Polish name instead of a Swedish name in the Swedish context. We argue that this is not simply due to the longer cognitive processing of the foreign-sounding name or foreign words in general (e.g., Mishra, 2009; Crossley et al., 2014), as in this case, we should not see any differences in the dwell-time on the face (race AOI) but rather on the name (ethnicity AOI). Additionally, the analysis showed that CV ratings were significantly different between Chinese (having a higher rating) and Iraqi names. This finding is in line with previous studies showing a higher level of discrimination for Middle Easterners (Agerström and Rooth, 2009; Rooth, 2010), and point to the ethnic hierarchies in the labor market. This differences across ethnic groups in rating is also interesting in relation with how race and ethnicity might have different meanings on the CV. Asian and Middle Eastern CVs were proportionally equally advanced to the top three selection, and in fact 3 Middle Eastern CVs whereof two were matched with a Swedish name were chosen as the top candidate, while only 1 Asian CV with a Swedish name was chosen as the top candidate.

In contrast to earlier research on hiring discrimination, we found that non-White CVs were generally more favorably rated than White CVs and thus had to reject our first hypothesis. How do we understand the current result together with the results that showed no significant correlation between dwell-time and CV rating? We suggest that our findings do not show the same effect because our respondents were professional respondents who were fully aware of what they were supposed to look for (Lahey and Oxley, 2018; Petersheim et al., 2022), and were all working for companies actively engaged in diversifying their organization. Additionally, we suggest that parts of the results can be explained by social desirability. Indeed, social desirability bias in attitudes toward diversity, equity and inclusion policies are observed in international studies, warning for the overestimate of support for diversity and inclusion in the workplace (e.g., Boring and Delfgaauw, 2023). Social desirability is about wanting to convey a positive self-description that is in line with organizational norms (Braun et al., 2001). This means that respondents were influenced by the wish to come across as non-discriminatory, as diversity and inclusion are organizational norms their organizations strive toward. Previous research from the Swedish context also shows that social desirability among workers in organizations is related with increased levels of color-blindness, where color-blindness can be understood as a way to appear unbiased (Penner and Dovidio, 2016; Schütze, 2023). Color-blindness and social desirability co-occur when respondents want to preserve a positive self-image and fit into the current social and organizational norms (Knowles et al., 2009).

Another aspect to consider related to color-blindness is the potential of aversive racism (Dovidio et al., 2017). Aversive racism is a racial bias that may be held by people who are politically and socially liberal, people who support racial equality and regard themselves as non-prejudiced (Nail et al., 2003). Studies within psychology indeed show that in some cases aversive racism is reflected in the expression of more positive feelings toward racial minority compared to their own White groups, and how this positivity toward racial diversity may obscure self-recognition of prejudice, which may surface in other instances, often unconsciously (Greenwald and Pettigrew, 2014). Aversive racism may influence selection decisions in employment, for example previous study shows that there is no discrimination against candidates with clearly strong qualification while when the qualification is ambiguous the discriminatory patterns emerge (Dovidio and Gaertner, 2000; Son Hing et al., 2008). Indeed, our design of the vignette study provided respondents with the instruction that all candidates are qualified for the job, which may have heightened a pro-diversity choice for respondents.

Considering social desirability and other related aspects discussed above, we need to triangulate the eye-tracking data, ratings and the dialog data in the future to evaluate this result carefully, and further analyze the gap between the rating and the actual behavior. Moreover, we need to examine the pattern of CV selection with a larger dataset to validate the patterns we observe in the current small scale experimental study.^8^ While not the focus of our study, we also find that respondents chose men and women equally. This may not be surprising considering the advancement in gender equality policies in Sweden and the occupational sector already seeing gender equality in numbers. In Sweden, financial assistant and accounting economists are among the top 20 female dominated occupation.^9^ When we look at more specific occupational categories, we also see that about 50% of managers in banking, finance and insurance, accountants, financial analysts, and fund managers, etc. and economic and financial managers are female.^10^

The analysis of the dialog shows the complexity of assessment of whether and how candidates fit to the organization. As previous studies suggest, there is individual variability in interpreting person-organization fit from information on qualification such as previous work experiences. We especially noted how respondents assumed person-organization fit through the limited information given on the CV, confirming previous studies that suggest a structured form of recruitment processes to eliminate as much bias and discrimination as possible (Noon, 2012; Wolgast et al., 2017; Feng et al., 2020; Rivera, 2020). Moreover, dialog shows that when the respondents discuss diversity in the organization, they pay more attention to gender (female) and a Black CV and not to other types of diversity such as non-Black racial groups and ethnicity, which might be explained by social desirability as discussed above. The dialog we analyzed was carried out in a controlled experimental situation. Future research is needed to understand how the consensus making process affects the understanding of who fits the job and organization, together with an analysis of whether there are unconscious bias such as aversive forms of racism that plays into the selection process, in a real-life recruitment situation.

Eye-tracking presents a potential in measuring attribute salience and attention discrimination. Moreover, while other parts of the study might have been affected by social desirability (as discussed above) and knowledge of the respondents that they were participating in the experiment addressing diversity and inclusion in hiring process, eye-movements cannot be consciously controlled. Eye-tracking is therefore one way to attenuate the social desirability that can be observed in survey response biases (Jenke et al., 2021). Previous studies indeed show that respondents are not affected by the social desirability caused by the usage of eye-trackers (Kee et al., 2021). In the dialog, the respondents spent very little time focusing on the aspects of racial, ethnic and gender diversity of the candidates in the consensus making dialog can be interpreted in two ways, which could be deriving from social desirability needs and expressions of aversive racism as discussed above. It can be the avoidance to talk about and address the actual lack of diversity in their workplace; in this case this result may be in line with the critique of diversity initiatives not leading up to more representation of diversity in the organization. If there was an avoidance to talk about racial and ethnic diversity, this may indeed be embedded in the Swedish color-blindness (Schütze and Osanami Törngren, 2022).

We acknowledge the research which shows that engagement with diversity initiatives does not necessarily lead to actual diversity and equity in the workplace, and the positively expressed attitudes might lead to overestimation of the actual change that may happen in an organization. Research also address the importance of managers in organizations taking accountability (Dobbin and Kalev, 2022). This study focuses on the individual choices of recruiters, and therefore it remains of crucial interest to understand how individuals directly working with hiring in these companies approach diversity and how they screen CVs.

The overall results of the paper have implications for our knowledge on hiring discrimination. Indeed, in our sample of companies looking to increase diversity, we do not find the classical pattern of hiring discrimination as was found in earlier studies. Nevertheless, we still find evidence of ethnic hierarchies, where Asian candidates are overall preferred to Middle-Easters. Our results show that these differences are not necessarily explained by differences in how employers scrutinize the CVs or attention discrimination. Therefore, it seems to be unlikely that small interventions on the style and layout of the CV will be an easy fix for the hiring discrimination many minority candidates still face in Sweden. Future research should shed light on which policy interventions governments and companies can make to assure that the future workforce is a true representation of the diverse societies we live in.

## Data availability statement

The datasets presented in this article are not readily available because of the Swedish research ethics regulations. Requests to access the datasets should be directed to sayaka.torngren@mau.se, however we cannot provide access to the dataset.

## Ethics statement

The studies involving humans were approved by the Swedish Ethical Review Authority (2022-00584-01). The studies were conducted in accordance with the local legislation and institutional requirements. The participants provided their written informed consent to participate in this study.

## Author contributions

ST was the project leader. CS was leading the analysis on the vignette. MN on the eye-tracking data and ST on the qualitative material. EV read the manuscript and was involved in the further analysis, writing and revision of the manuscript. All authors were equally involved in the design and execution of the project.

## Footnotes

^1^SCB: https://www.scb.se/hitta-statistik/statistik-efter-amne/befolkning/befolkningens-sammansattning/befolkningsstatistik/.

^2^EUROSTAT: https://ec.europa.eu/eurostat/statistics-explained/index.php?title=Foreign-born_people_and_their_descendants_-_educational_attainment_level_and_skills_in_host_country_language; https://ec.europa.eu/eurostat/statistics-explained/index.php?title=Foreign-born_people_and_their_descendants_-_labour_market_conditions.

^3^The project collaborate with a diversity consultancy company which initially facilitated the contacts with key persons in the company.

^4^The survey was programmed in Qualtrics. We have implemented the IAT utilizing the open-source provided in Carpenter, T. P., Pogacar, R., Pullig, C., Kouril, M., Aguilar, S., Labouff, J., Isenberg, N. and Chakroff, A. 2019. Survey-software implicit association tests: A methodological and empirical analysis. *Behavior research methods,* 51, 2,194–2,208.

^5^MA, D. S., Kantner, J. & Wittenbrink, B. 2021. Chicago face database: Multiracial expansion. *Behavior Research Methods*, 53, 1,289–1,300, Saribay, S. A., Biten, A. F., Meral, E. O., Aldan, P., Třebický, V. and Kleisner, K. 2018. The Bogazici face database: Standardized photographs of Turkish faces with supporting materials. *PloS one*, 13, e0192018.

^6^From our initial list of names, we selected the names to be included in our experiment, excluding names that were perceived to be older than 35, names that were associated with a low or high social class, names that were rated as very unattractive or very attractive and names that were perceived to be very religious. We had to make several exceptions here, as all our Polish names were perceived to be older than 35 and the majority of the Chinese names were perceived to be of lower social class. From the remaining names, we selected these names that were most likely to be assigned the correct ethnicity and the correct gender.

^7^ https://www.reed.edu/psychology/stata/analyses/parametric/ANOVA/tukey.html

^8^Larger data collection through online vignette survey is ongoing.

^9^ https://www.scb.se/hitta-statistik/statistik-efter-amne/arbetsmarknad/sysselsattning-forvarvsarbete-och-arbetstider/yrkesregistret-med-yrkesstatistik/pong/tabell-och-diagram/20-vanligaste-yrkena-for-kvinnor/

^10^Employees aged 16–64 in the country by occupation (3-digit SSYK 2012), industry SNI2007 (rough level), age and sex. New time series. Year 2019–2021: https://www.statistikdatabasen.scb.se/pxweb/sv/ssd/START__AM__AM0208__AM0208B/YREG61N/.
