# Supplementary material for: “We choose this CV because we choose diversity” – What do eye movements say about the choices recruiters make?
Source: Front Sociol. 2024 Mar 7;9:1222850. doi: 10.3389/fsoc.2024.1222850 (PMC10954785; doi:10.3389/fsoc.2024.1222850)
Supplement: Supplementary file 1 [file Table_1.pdf]

Appendix

Table 1. CVs in vignette studies per race and ethnicity

	TOTAL (N=200)	Top 3 (N=100)	Top candidate (N=20)
Asian-Swedish name	8	3	1
Asian-Chinese name	15	8	0
Black-Swedish name	6	4	2
Black-Eritrean name	21	17	7
Middle East-Swedish name	17	7	1
Middle East-Iraqi name	5	5	2
White-Swedish name	79	31	4
White-Polish name	49	25	3

Table 2. List of names that were used in the CVs

**Male**	**Female**	**Male**	**Female**
**Swedish**		**Eritrean**	
Albin Andersson	Tilda Johansson	Afwerki Berhane	Leah Tekle
Felix Axelsson	Johanna Larsson	Haile Yemane	Niyat Solomon
Anton Bergström	Viktoria Lindberg	**Chinese**	
Erik Eriksson	Therese Lindström	Cheng Xiao	Liu Hua
Oskar Fredriksson	Emma Lundgren	Li Yuxuan	Sun Ying
Hampus Gustafsson	Klara Lundqvist	**Iraqi**	
Viktor Hansson	Amanda Magnusson	Hassan Haidar	Amina Saif
Filip Henriksson	Elsa Olofsson	Ibrahim Hussein	Nidal Ali
Jakob Jakobsson	Frida Persson	**Polish**	
Jonathan Jansson	Saga Svensson	Krzysztof Kozlowski	Elżbieta Pawlowski
		Patryk Kwiatkowski	Teresa Dabrowski

Table 3. Example of quotes for each coded category

**Qualification**	
Work experience	Everyone seems to have worked in a store as well as customer client facing. But I think that when you are so early in your career, it is only good that they have worked rather than what they have worked with.
Skills	I think it says Microsoft Excel or Microsoft Office on all of them. And for me, yes, Microsoft Excel is Office and Excel is probably the most important thing for the economist, I think.
Education	I made the decision solely on that [education], so the ones I chose to call in [for an interview] were more likely those who had a master's degree and that it says passed with distinction.
**Values and social skills**	
Company values and interests	"Econometric approach on a new index of welfare." It's so far away from our business and what you might think is interesting. (Thesis topics)<br><br>We now say the number of years as something positive, but at the same time we will also expect that they may have received slightly more responsible assignments if they have been in the same position for a longer time. (Type of work)
Motivation	"Exciting career as an economist with recently acquired degree in Business and Economics" So yes. I believe in this one. I think she is the most forward-looking in this [motivation section] also, "I want to have and I look forward to an exciting career" while the other one is "I want to develop myself."
Social skills	And there [customer service on the phone], you really must dare to have a cold conversation. You don't need that in our organization, but you learn to deal with clients in a good way often.
**Diversity**	I thought OK I need to go for a bit of diversity, so I picked the other girl [Middle Eastern Female]. Just because I often try to promote girls into workplaces. And I picked an Asian looking candidate.
